# Value of sonoelastography for diagnosis of breast non-mass lesions and comparison with BI-RADS: A systematic review and meta-analysis

**DOI:** 10.1097/MD.0000000000038425

**Published:** 2024-06-07

**Authors:** Hong Li, Peng Cong, Ya-Na Yu, Yun-Fei Zhang

**Affiliations:** a Department of Transplantation and General Surgery, The First Hospital of China Medical University, Shenyang, China; b Department of Ultrasound, The First Hospital of China Medical University, Shenyang, China.

**Keywords:** BI-RADS, breast, meta-analysis, non-mass, sonoelastography

## Abstract

**Background:**

Not all the breast lesions were mass-like, some were non-mass-like at ultrasonography. In these lesions, conventional ultrasonography had a high sensitivity but a low specificity. Sonoelastography can evaluate tissue stiffness to differentiate malignant masses from benign ones. Then what about the non-mass lesions? The aim of this study was to evaluate the current accuracy of sonoelastography in the breast non-mass lesions and compare the results with those of the American College of Radiology breast Imaging-Reporting and Data System (BI-RADS).

**Methods:**

An independent literature search of English medical databases, including PubMed, Web of Science, Embase & MEDLINE (Embase.com) and Cochrane Library, was performed by 2 researchers. The accuracy of sonoelastography was calculated and compared with those of BI-RADS.

**Results:**

Fourteen relevant studies including 1058 breast non-mass lesions were included. Sonoelastography showed a pooled sensitivity of 0.74 (95% CI: 0.70–0.78), specificity of 0.89 (95% CI: 0.85–0.91), diagnostic odds ratio (DOR) of 25.22 (95% CI: 17.71–35.92), and an area under the curve of 0.9042. Eight articles included both sonoelastography and BI-RADS. The pooled sensitivity, specificity, DOR and AUC were 0.69 versus 0.91 (*P* < .01), 0.90 versus 0.68 (*P* < .01), 19.65 versus 29.34 (*P* > .05), and 0.8685 versus 0.9327 (*P* > .05), respectively.

**Conclusions:**

Sonoelastography has a higher specificity and a lower sensitivity for differential diagnosis between malignant and benign breast non-mass lesions compared with BI-RADS, although there were no differences in AUC between them.

## 1. Introduction

According to the American College of radiology breast imaging-reporting and data system (BI-RADS) lexicon for breast ultrasound (US), a mass is defined as a space-occupying lesion seen in 2 different planes and can be distinguished from normal anatomic structures.^[1]^ However, not all breast lesions strictly meet the criteria described by BI-RADS, such as tubular hypoechoic duct-like structures, ill-defined geographic hypoechoic lesions, or architectural distortion.^[2,3]^ Symptoms of these non-mass lesions include palpable breast mass, breast pain, nipple discharge, etc. These clinical manifestations lack specificity, making them susceptible to being misdiagnosed or missing a diagnosis. It has been reported that conventional US has a high sensitivity of 95.35% but a low specificity of 43.24% in the diagnosis of non-mass lesions.^[3]^ The unsatisfactory results are probably due to the overlap in US features between malignant and benign breast non-mass lesions, which included ductal carcinoma in situ (DCIS), intraductal carcinoma, invasive lobular carcinoma (ILC), fibrosis, and fibrocystic changes.^[3–7]^ For patients with suspected breast lesions, biopsy is usually recommended to confirm the diagnosis, but studies have shown that the histological consistency between US-guided core-needle biopsy (US-CNB) and surgical histological diagnosis was significantly better in mass lesions than in non-mass lesions.^[3]^ Moreover, according to reports, 53.8% to 72.7% of breast non-mass lesions were benign,^[8,9]^ and excessive reliance on biopsy may increase the psychological and economic burden on these patients. Therefore, more technology is required to offer valuable information for the identification of breast non-mass lesions, aiding doctors in developing appropriate treatment regimens.

Sonoelastography is an innovative US technique that evaluates tissue stiffness based on the principle that malignant tissues are generally harder than benign tissues.^[6]^ Recently, many studies have examined the diagnostic accuracy of sonoelastography for differential diagnosis between malignant and benign breast masses, which showed a good sensitivity of 85.5% to 88.8% and specificity of 84.8% to 90.2%.^[10–12]^ However, few research has focused on breast non-mass lesions. Some studies have shown that sonoelastography has important value in the diagnosis of breast diseases, especially for non-mass lesions.^[13,14]^ The aim of our study was to summarize the diagnostic performance of sonoelastography for breast non-mass lesions and determine the application value of this technology.

## 2. Materials and methods

All analyses were based on previously published studies; thus, no ethical approval or patient consent were required.

### 2.1. Literature search

Two independent researchers performed the study in accordance with the PRISMA recommendations.^[15,16]^ The PubMed, Web of Science, Embase & MEDLINE (Embase.com), and Cochrane Library databases were searched to identify all studies that assessed differential diagnosis between malignant and benign breast non-mass lesions. Table 1 shows the search strategy. Duplicate articles were manually excluded. Unpublished relevant data were also considered, but no suitable studies were appropriate for inclusion. The literature search was updated until March 23, 2024.

**Table 1:** Search strategy of each database.

Database	Strategy
Pubmed	((((((((((“Elasticity Imaging Techniques”[Mesh]) OR elasticity imaging technique) OR tissue elasticity imaging) OR elastography) OR vibro acoustography) OR acoustic radiation force impulse) OR sonoelastography) OR elastograms)) AND (((((“Breast”[Mesh]) OR “Mammary Glands, Human”[Mesh])) OR Breast) OR mammary)) AND Non mass
Embase & medline (Embase.com)	(#1) elasticity AND imaging AND technique OR (tissue AND elasticity AND imaging) OR elastography OR (vibro AND acoustography) OR (acoustic AND radiation AND force AND impulse) OR sonoelastography OR elastogram (#2) breast OR mammary (#3) non mass (#4) #1 AND #2 AND #3
Cochrane library	(#1) Mesh descriptor: [Elasticity Imaging Techniques] explode all trees (#2) elasticity imaging technique OR tissue elasticity imaging OR elastography OR vibro acoustography OR acoustic radiation force impulse OR sonoelastography OR elastogram (Word variations have been searched) (#3) #1 OR #2 (#4) Mesh descriptor: [Breast] explode all trees (#5) Mesh descriptor: [Mammary Glands, Human] explode all trees (#6) breast OR mammary (Word variations have been searched) (#7) #4 OR #5 OR #6 (#8) non mass (Word variations have been searched) (#9) #3 AND #7 AND #8
Web of science	TOPIC: ((elasticity imaging technique) OR (tissue elasticity imaging) OR (elastography) OR (vibro acoustography) OR (acoustic radiation force impulse) OR (sonoelastography) OR (elastogram)) AND TOPIC: ((breast) OR (mammary)) AND TOPIC: (non mass)

### 2.2. Inclusion and exclusion criteria

Two researchers assessed the articles independently. The inclusion criteria were as follows: the study was approved by an ethics committee or institutional review board; sonoelastography was performed for the breast non-mass lesions in the study; surgery, US-CNB, and/or US-guided vacuum-assisted biopsy (US-VAB) results were used as the reference standard in the study; reported data were necessary to identify the true positive (TP), false positive (FP), false negative (FN), and true negative (TN) cases. The exclusion criteria were as follows: case reports, letters, reviews, editorial comments, conference reports, and articles that were not published in English; in the presence of insufficient data, the corresponding authors were contacted via email to request missing data, but there was no response within 15 days; in cases where 2 or more studies were performed by the same department in an overlapping period, studies with a smaller number of patient samples were excluded. All the disagreements were resolved by consensus.

### 2.3. Data extraction

The data were extracted by 2 investigators independently. All relevant data from the included studies were extracted, including first author, country where the study was performed, publication year, patient age, symptoms, number of patients, number of lesions, lesion size, reference standard, type of lesions, US system that was used in the study, index of elastography, cutoff value, and number of TPs, FPs, FNs, and TNs. In articles including BI-RADS, the TPs, FPs, FNs, and TNs of BI-RADS were also acquired. The Youden method was used to define the cutoff value if the value was not clearly provided by the author. All the disagreements were resolved by consensus.

### 2.4. Quality assessment

Methodological quality was assessed using the quality assessment of diagnostic accuracy studies criteria.^[17]^ The defined questions were answered as yes, no, or unclear and were assigned 1 point, 0 point, and 0.5 point, respectively. All the items in the questions were completed by 2 researchers independently. Disagreements were resolved by consensus.

### 2.5. Data analysis

The statistical software STATA (Version 18.0, Stata Corporation), Meta-Disc (Version 1.4, Unit of Clinical Biostatistics team of the Ramóny Cajal Hospital), and SPSS Statistics (Version 25.0, IBM Corporation) were used in the study. The threshold effect was analyzed with the Spearman correlation coefficient. Other heterogeneities were evaluated by the Cochran *Q* statistic and the *I*^2^ test of the diagnostic odds ratio (DOR). Meta-Disc software was used to calculate the pooled sensitivity, specificity, DOR, area under the curve, and Q* index. STATA software was used to generate Deek’s funnel plot and analyze the potential publication bias, with a *P* value* < *.05 indicating potential publication bias. SPSS software was used to analyze interobserver agreement with Cohen’s κ while screening articles and applying the quality assessment of diagnostic accuracy studies criteria. The *Z* test was used to compare pooled sensitivity, specificity, DOR, and AUC between sonoelastography and BI-RADS.

## 3. Results

### 3.1. Literature search and characteristics of the included studies

**Figure 1.**
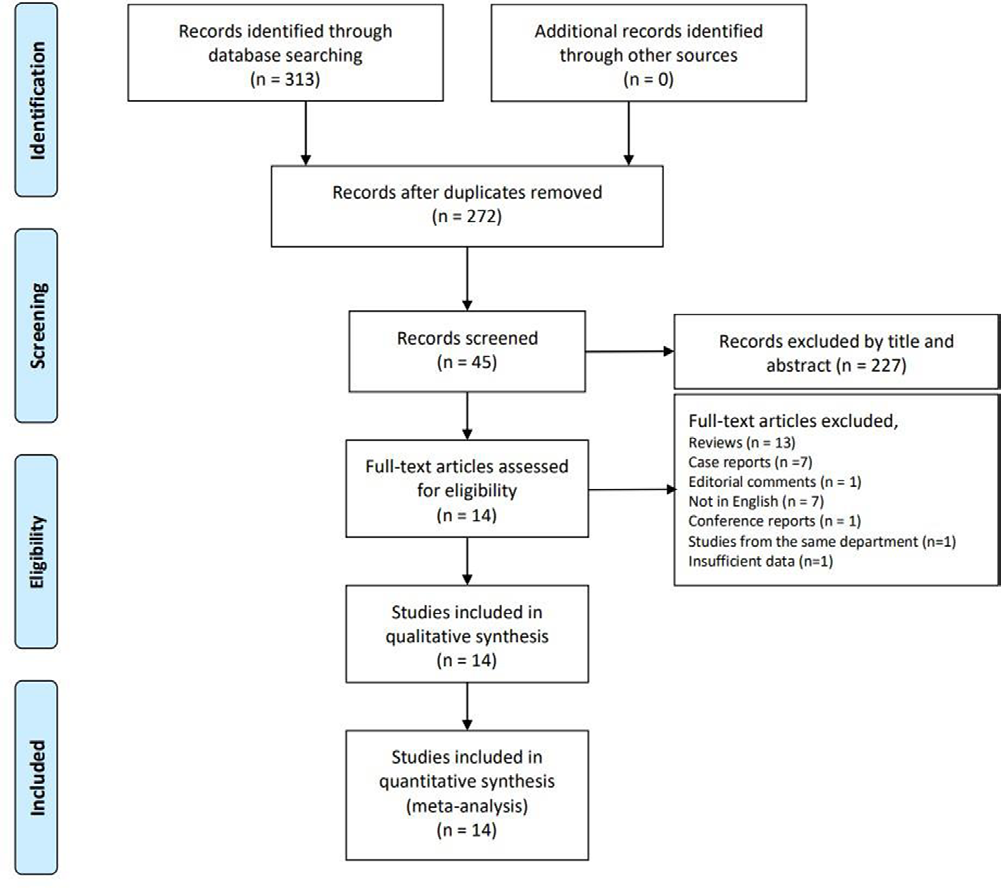
The research selection and literature retrieval process of this paper (a total of 10 studies).

Fourteen related studies, including 1058 breast non-mass lesions, were included in the meta-analysis after a literature search, which were published from 2012 to 2023^[6,7,13,18–28]^ (Fig. 1). Table 2 shows the main characteristics of the included studies. In one study with insufficient data, the missing data were provided by the corresponding author via email.^[22]^ There were 2 studies from the same department: one was performed between April 2013 and February 2016 and included 85 breast non-mass lesions,^[19]^ and the other was performed from December 2014 to November 2016 and included 82 lesions.^[29]^ Thus, the latter article was excluded. Excellent interobserver agreement was observed when the studies were excluded by title and abstract (κ = 0.881). In other steps, there was no disagreement between the 2 observers (κ = 1).

**Table 2:** Main characteristics of included studies.

	Author	Country	Year	Age (range, avg. or median: yrs)	Symptoms, (number of patients, %)	Number of patients	Number of lesions	Lesion Size (range, mean diameter: mm)	Reference standard	Type of lesions (number of lesions)	Ultrasound system	Index of elastography	Cutoff value	TP	FP	FN	TN
1	Ko ES et al^[6]^	Korea	2012	NA	NA	NA	36	NA	Malignancy: surgical excision and US-guided CNB; benign: US-guided CNB and follow-up	Invasive ductal carcinoma (13), DCIS) (5), invasive lobular carcinoma (ILC) (1), invasive papillary carcinoma (1), lobular carcinoma in situ (1); fibrocystic change (6), papilloma (2), fibrosis (2), benign mastopathy (2), adenosis (1), inflammatory lesions (2)	Hitachi medical, EUB-7500	SE (5-point)	SE4	12	0	9	15
2	Ko KH et al^[7]^	Korea	2014	36–62, 46.4	Palpable breast mass (15, 45%), nipple discharge (1, 3%), and breast pain (3, 9%)	33	34	4–50, 25.4	US-guided automated gun biopsy (11) or US-guided vacuum-assisted biopsy (5) or surgical excision (18)	Infiltrating ductal carcinoma (6), DCIS (4), lobular carcinoma in situ (2); fibrocystic change (6), fibroadenomatous hyperplasia (5), stromal fibrosis (7), intraductal papilloma (2), chronic inflammation (1), adenosis (1)	Supersonic Imagine, Aixplorer system	Emean	41.6 kPa	10	7	2	15
3	Choi JS et al^[18]^	Korea	2016	29–85, 48.4	NA	113	116	6–60, 27.5	Surgery (74 malignant and 7 benign); US-CNB and US-VAB (2 benign); US-CNB and follow-up (33 benign)	Invasive ductal carcinoma (36), microinvasive ductal carcinoma (5), DCIS (19), ILC (10), invasive micropapillary carcinoma (2), metaplastic carcinoma (1), breast metastasis from ovarian cancer (1); stromal fibrosis (10), fibrocystic change (8); fibroadenoma (5), fibroadenomatoid mastopathy (4), granulomatous lobular mastitis (4), intraductal papilloma (2), sclerosing adenosis (2), atypical ductal hyperplasia (1), flat epithelial atypia (1), apocrine metaplasia (1), adenosis (1), columnar cell change (1), duct ectasia (1), fat necrosis (1)	Supersonic imagine, aixplorer system	Emean	85.1 kPa	58	2	16	40
4	Li L et al#,^[19]^	China	2017	18–78, 44.5	Palpable mass (72, 92%), nipple discharge, and palpable mass (2, 3%)	77	77	7.4–51.9, 16.9	US-CNB (All); Surgery; US-CNB and follow-up (benign)	Infiltrating ductal carcinoma (25), DCIS (17), solid papillary carcinoma (2), and ILC (1); fibrocystic change (11), fibroadenoma (10), papilloma (3), chronic inflammation (3), radial scar (2), sclerosing adenosis of the breast (1), atypical ductal hyperplasia (1)	Hitachi medical, hitachi HV-900 or EUB-8500	SE (5-point)	SE4	39	7	7	24
5	Park SY et al^[20]^	Korea	2017	25–85, 47	NA	147	152	5–60, 20	Surgery and US-CNB (79 malignant, 13 benign); US-CNB and US-VAB (5 benign); US-CNB and follow-up (55 benign)	DCIS (21), invasive ductal carcinoma (46), ILC (10), metaplastic carcinoma (1) and mucinous carcinoma (1); papillary lesion (6), atypical ductal hyperplasia (2), flat epithelial hyperplasia (1), stromal fibrosis (18), fibrocystic change (13), sclerosing adenosis (8), fibroadenomatoid mastopathy (7), fibroadenoma (6), granulomatous lobular mastitis (4), columnar cell change (3), fat necrosis (2), apocrine metaplasia (1), diabetic mastopathy (1), usual ductal hyperplasia (1)	Supersonic imagine, aixplorer system	Emean	85.1 kPa	54	5	25	68
6	Wang, ZL et al^[21]^	China	2017	23–65, 40.1	Palpable breast lesion (15, 23.8%), nipple discharge (2, 3.2%), and breast pain (11, 17.4%)	63	67	11–105, 41.4	US-CNB (22), US-VAB (10), or surgery (35)	Infiltrating ductal carcinomas (16), DCIS (13), ILC (1), lymphoma (1) and papillocarcinoma (2); fibroadenoses (23), Papillomas (4), inflammations (4), fibroadenomas (3)	Philips medical systems, iU22 ultrasound system	Emean	63.71 kPa	19	2	14	32
7	Graziano L et al*,^[22]^	Brazil	2017	NA	NA	NA	27	NA, 23	Percutaneous or surgical biopsy	Invasive carcinoma (no special type) (4), DCIS (5), tubular carcinoma (1); stromal fibrosis (8), papilloma (3), fibrocystic changes (6)	Toshiba America medical systems, Aplio 500	SE (5-point)	SE4	8	3	2	14
8	Qu, XX et al^[23]^	China	2019	22–67, 49.4	Palpable breast mass (21, 53.8%), breast pain (14, 35.9%), and both breast pain and palpable breast mass (4, 10.3%)	39	39	NA	Subsequent core-needle biopsy or surgical excision	DCIS or with micro-infiltration (4), DCIS mostly with invasive ductal carcinoma (6), invasive ductal carcinoma partly with DCIS (6), invasive ductal carcinoma (5), intraductal papillary carcinoma partly with DCIS (1), metastatic carcinoma (1); fibrocystic changes (7), fibrocystic changes with atypical hyperplasia (3), adenosis (3), plasma cell mastitis (2), chronic inflammation with micro-abscess (1)	Hitachi medical, EUB-7500, and HIVISION preirus scanner	SR	4.07	21	1	2	15
9	Xu, P et al^[25]^	China	2020	Benign (NA, 49.70), malignant (NA, 50.56)	NA	118	118	Benign (NA, 21.0), malignant (NA, 23.8)	Pathological results	Intraductal carcinoma in situ (30), invasive ductal carcinoma (19), ILC (2), lymphoma (1); adenosis (32), intraductal papilloma (9), breast inflammation (8), atypical ductal hyperplasia (7), fibroadenoma (6), sclerosing adenosis (4)	Mindray, ultrasonic system resona 7	E2.5 max	94.62 kPa	49	9	3	57
10	Sefidbakht S et al^[24]^	Iran	2021	28–75, 47.5	Palpable breast mass (23, 47%)	49	49	NA	Core-needle biopsy	DCIS (5), invasive carcinoma (7); non-high-risk benign lesions (32), high-risk benign lesions (5)	Supersonic, aix-en-provence	Maximum color	Green to yellow	7	4	5	33
11	Guo, WJ et al^[26]^	China	2022	26–88, 51.91	Palpable mass (50, 50%), nipple discharge (15, 15%), and breast pain (20, 20%)	100	101	4–56, 21.05	Surgery or biopsy	DCIS (17), invasive ductal carcinoma (16), invasive ductal carcinoma + DCIS (9), intraductal papillary carcinoma (4), solid papillary carcinoma (2), mucinous breast carcinoma (1), lobular carcinoma in situ (1), Paget’s disease + DCIS (1); adenosis (18), intraductal papillo.ma (14), granulomatous mastitis (8), mammary duct ectasia (5), fibroadenoma (3), sclerosing adenosis (2)	Aplio i900 system	SE (5-point)	SE4	35	8	16	42
12	Li, JN et al^[13]^	China	2023	NA, 48.8	NA	NA	115	Benign (NA, 20), malignant (NA, 30)	Histopathological results and a minimum follow-up of 1 yr	NA	GE LOGIQ E20	Emax	196.3 kPa	36	5	24	50
13	Wang, FX et al^[27]^	China	2023	21–70, 44.4	Localized pain, and the corresponding signs include localized hardening and poor mobility of the breast tissue (some), no specific symptoms (most)	60	60	Benign (NA, 28.7), malignant (NA, 27.1)	US-guided core-needle biopsy, spin biopsy, or surgery	DCIS (12), invasive ductal carcinoma (8), DCIS combined invasive ductal carcinoma (6), ILC (4), invasive micropapillary carcinoma (1), mucinous carcinoma (1), lymphoma (1); fibroadenoma (9), fibrocystic mastopathy (13), intraductal papilloma (3), plasma cell mastitis (1), granulomatous mastitis (1)	Supersonic aixPlorerV imaging instrument	Emax, Emean or Emin; the elastic ratio of lesion/surrounding normal tissue; malignant signs	Emax ≥ 48.3 Kpa, Emean ≥ 33.3 Kpa or Emin ≥ 20.9 KPa; the elastic ratio of lesion/surrounding normal tissue ≥ 2.4; stiff-rim sign, multi-colored sign, void center sign, and horseshoe sign were all considered malignant presentations	25	4	8	23
14	Kayadibi, Y et al^[28]^	Turkey	2023	35–73, NA	NA	67	67	Benign (NA, 17), malignant (NA, 24.7)	US-guided core needle biopsy and surgical specimens	DCIS (24), invasive ductal carcinomas (21); fibroadenomatoid development (5), severe epithelial hyperplasia (4), mild epithelial hyperplasia (3), sclerosing adenosis (2), fat necrosis (4), adenosis-fibrosis (3), atypical lobular hyperplasia (1)	Toshiba Aplio A US machine	Emean	38 Kpa	35	1	10	21

Avg. = average, CNB = core needle biopsy, DCIS = ductal carcinoma in situ, FN = false negative, FP = false positive, ILC = invasive lobular carcinoma, NA = not available, SE = strain elastography, TN = true negative, TP = true positive, US = ultrasound, VAB = cacuum-assisted biopsy.

# another study from the same department was omitted.

* missing data provided by corresponding author via e-mail.

### 3.2. Quality assessment

Table 3 shows the quality assessment of each study. All the included studies scored at least 13 points, indicating that they were “high” quality studies. However, in 13 of the 14 included studies, it was unclear whether pathologists were blinded to the sonoelastography results. In 2 studies, it was unclear if readers of sonoelastography were blinded to the histological results. The interobserver agreement was excellent between the 2 researchers (κ = 0.816).

**Table 3:** Quality assessment of the included studies using the “QUADAS” questionnaire.

QUADAS questionnaire	Ko ES et al^[6]^	Ko KH et al^[7]^	Choi JS et al^[18]^	Li L et al^[19]^	Park SY et al^[20]^	Wang, ZL et al^[21]^	Graziano L et al^[22]^	Qu, XX et al^[23]^	Xu, P et al^[25]^	Sefidbakht S et al^[24]^	Guo, WJ et al^[26]^	Li, JN et al^[13]^	Wang, FX et al^[27]^	Kayadibi, Y et al^[28]^
1: Was the spectrum of patient representative of the patients who will receive the test in practice?	Yes	Yes	Yes	Yes	Yes	Yes	Yes	Yes	Yes	Yes	Yes	Yes	Yes	Yes
2: Were selection criteria clearly described?	Yes	Yes	Yes	Yes	Yes	Yes	Yes	Yes	Yes	Yes	Yes	Yes	Yes	Yes
3: Is the reference standard likely to correctly classify the target condition?	Yes	Yes	Yes	Yes	Yes	Yes	Yes	Yes	Yes	Yes	Yes	Yes	Yes	Yes
4: Is the time period between reference standard and index test short enough to be sure that the target condition did not change between the 2 tests?	Yes	Yes	Yes	Yes	Yes	Yes	Yes	Yes	Yes	Yes	Yes	Yes	Yes	Yes
5: Did the whole sample, or a random selection of the sample, receive verification using a reference standard of diagnosis?	Yes	Yes	Yes	Yes	Yes	Yes	Yes	Yes	Yes	Yes	Yes	Yes	Yes	Yes
6: Did patients receive the same reference standard regardless of the index test result?	Yes	Yes	Yes	Yes	Yes	Yes	Yes	Yes	Yes	Yes	Yes	Yes	Yes	Yes
7: Was the reference standard independent of the index test (i.e., the index test did not form part of the reference standard?).	Yes	Yes	Yes	Yes	Yes	Yes	Yes	Yes	Yes	Yes	Yes	Yes	Yes	Yes
8: Was the execution of the index test described in sufficient detail to permit replication of the test?	Yes	Yes	Yes	Yes	Yes	Yes	Yes	Yes	Yes	Yes	Yes	Yes	Yes	Yes
9: Was the execution of the reference standard described in sufficient detail to permit replication?	Yes	Yes	Yes	Yes	Yes	Yes	Yes	Yes	Yes	Yes	Yes	Yes	Yes	Yes
10: Were the index test results interpreted without knowledge of the results of the reference standard?	Unclear	Yes	Yes	Unclear	Yes	Yes	Yes	Yes	Yes	Yes	Yes	Yes	Yes	Yes
11: Were the reference standard results interpreted without knowledge of the results of the index test?	Unclear	Unclear	Unclear	Unclear	Unclear	Yes	Unclear	Unclear	Unclear	Unclear	Unclear	Unclear	Unclear	Unclear
12: Were the same clinical data available when test results were interpreted as would be available when the test is used in practice?	Yes	Yes	Yes	Yes	Yes	Yes	Yes	Yes	Yes	Yes	Yes	Yes	Yes	Yes
13: Were un-interpretable/intermediate test results reported?	Yes	Yes	Yes	Yes	Yes	Yes	Yes	Yes	Yes	Yes	Yes	Yes	Yes	Yes
14: Were withdrawals from the study explained?	Yes	Yes	Yes	Yes	Yes	Yes	Yes	Yes	Yes	Yes	Yes	Yes	Yes	Yes
QUADAS score	13	13.5	13.5	13	13.5	14	13.5	13.5	13.5	13.5	13.5	13.5	13.5	13.5

### 3.3. Diagnostic performance of sonoelastography

**Figure 2.**
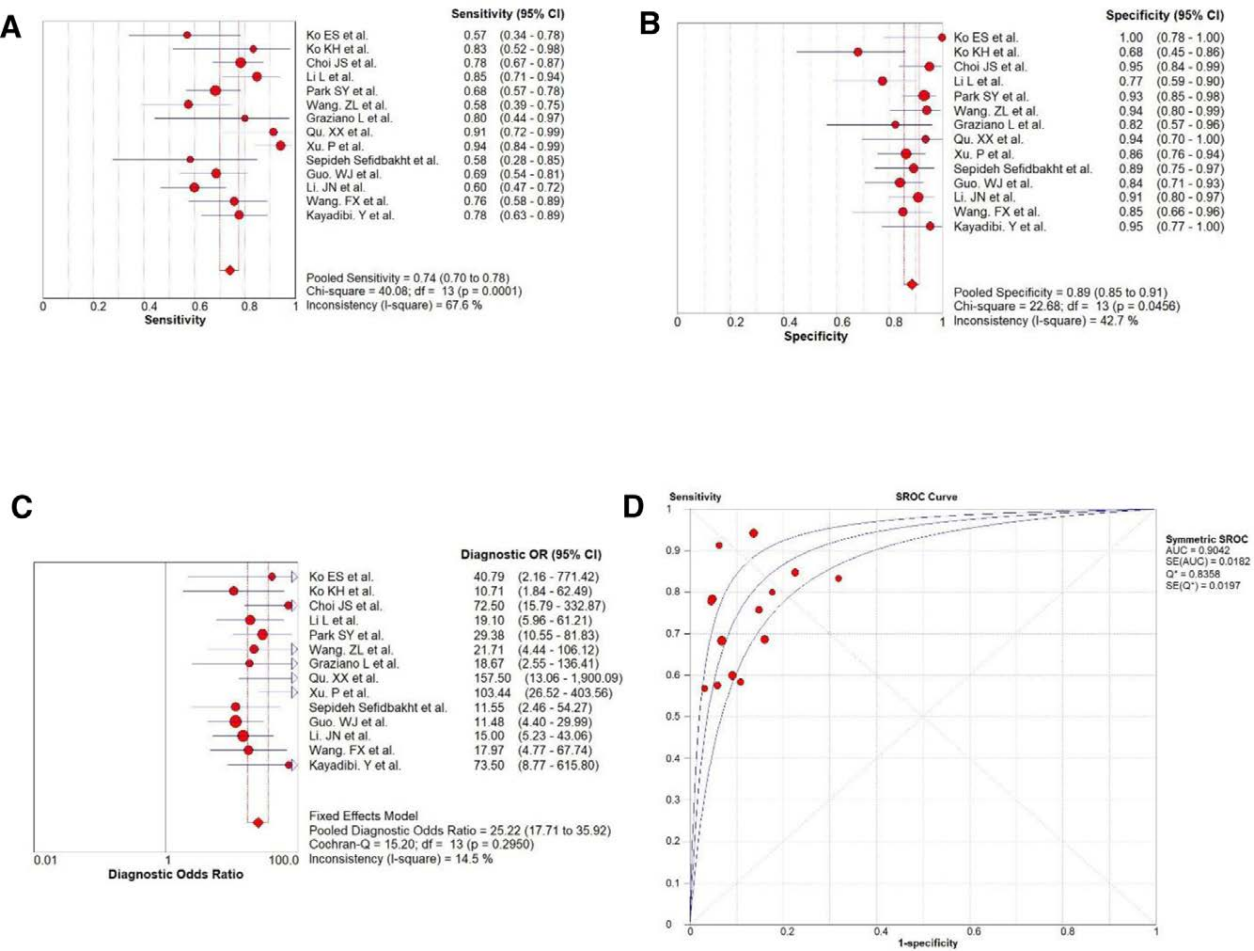
The Forest map results of sensitivity (A), specificity (B) and DOR (C) of sonoelastography for diagnosis and SROC curve of sonoelastography diagnostic accuracy obtained in this paper (D). DOR. DOR= diagnostic odds ratio, SROC = receiver operating characteristic.

The diagnostic performance of sonoelastography for differential diagnosis between malignant and benign breast non-mass lesions was computed based on a pooled sensitivity of 0.74 (95% CI: 0.70–0.78), specificity of 0.89 (95% CI: 0.85–0.91), and DOR of 25.22 (95% CI: 17.71–35.92). The summary receiver operating characteristic curve showed an AUC of 0.9042 (Fig. 2).

### 3.4. Comparison between sonoelastography and breast imaging-reporting and data system

**Figure 3.**
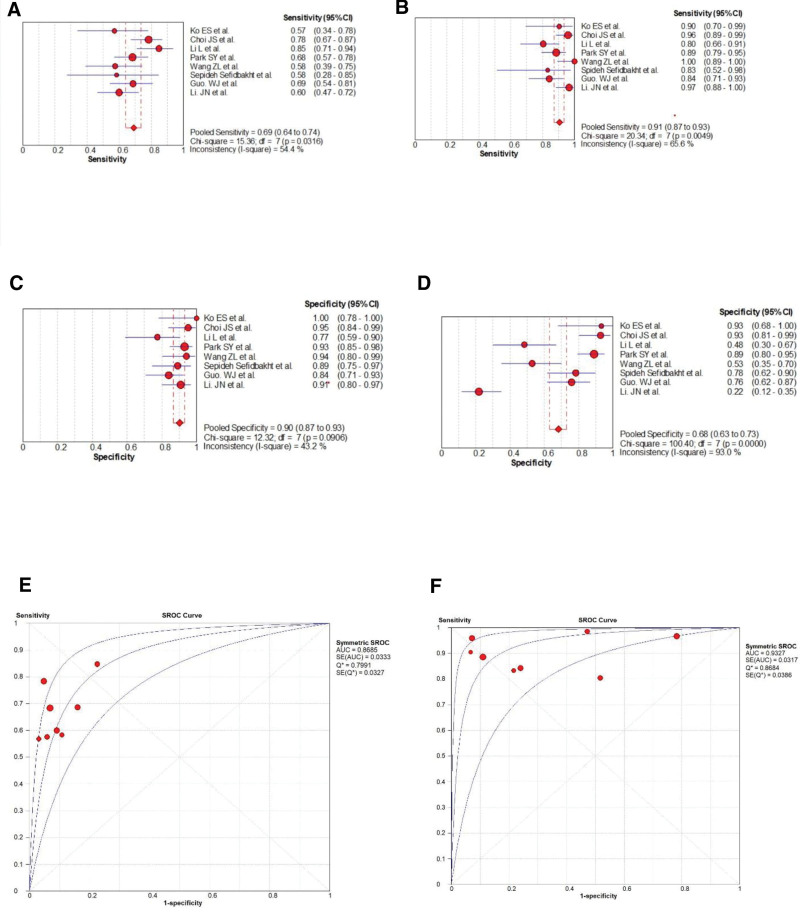
The Forest map results of sensitivity (A, B) and specificity (C, D) of sonoelastography and BI-RADS for diagnosis, respectively and summary receiver operating characteristic (SROC) curve of their diagnostic accuracy obtained in this paper (E, F). BI-RADS = breast imaging-reporting and data system, SROC = summary receiver operating characteristic.

There were 8 articles including both sonoelastography and BI-RADS. The cutoff value of BI-RADS was set at 4A because the highest Youden index was obtained when the value was over it in most of the articles. The pooled sensitivity, specificity, DOR and AUC were 0.69 versus 0.91 (*P* < .01), 0.90 versus 0.68 (*P* < .05), 19.65 versus 29.34 (*P* > .05), and 0.8685 versus 0.9327 (*P* > .05) in sonoelastography and BI-RADS, respectively (Fig. 3).

### 3.5. Heterogeneity assessment

Statistical analysis revealed no heterogeneity arising from the threshold effect (Spearman correlation coefficient: 0.455, *P* = .102). The Cochran *Q* test and the *I*^2^ test of DOR revealed no other heterogeneity with *P *= .295 and *I*^2^ = 14.5%.

### 3.6. Publication bias

**Figure 4.**
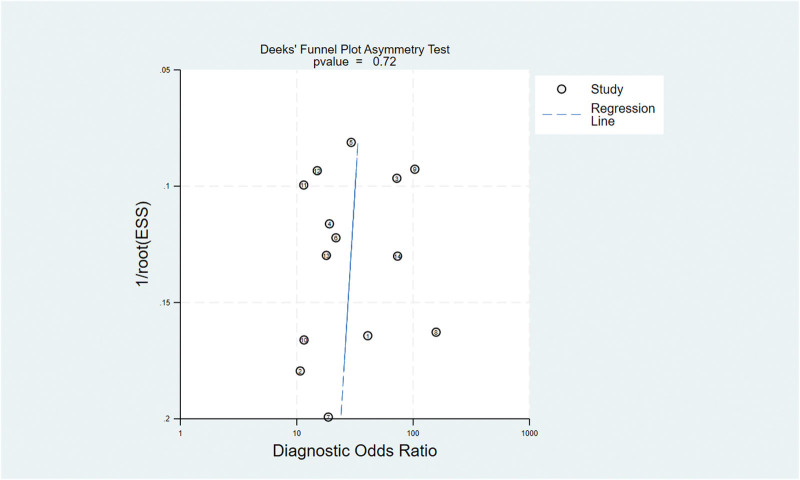
The results of Deek’s funnel plot: No significant bias was found.

Deek’s funnel plot was used to explore publication bias, which showed no significant differences in this meta-analysis (*P *= .716) (Fig. 4).

## 4. Discussion

Conventional US is widely used to identify or diagnose breast diseases. However, the differentiation of breast non-mass lesions by ultrasonography is still unclear, and there is significant overlap between the image features of malignant and benign lesions.^[26]^ Sonoelastography is an innovative ultrasonographic technique for assessing tissue stiffness. Recently, several original studies have focused on the value of sonoelastography for differential diagnosis between malignant and benign breast masses, which showed good performance.^[10–12]^ Among the possible debates surrounding sonoelastography, it should be included whether the method can be applied to non-mass lesions. Some authors described that there were no statistically significant differences in the diagnostic performance of sonoelastography between mass and non-mass lesions.^[6]^ Nevertheless, others described that the exclusion of non-mass lesions could improve their sensitivity and specificity values compared to other series and considered that interspersed healthy tissue could possibly lead to FN results.^[30]^

Our current meta-analysis showed a pooled sensitivity of 74% and a pooled specificity of 89% for differentiating between malignant and benign breast non-mass lesions. The specificity was similar to that of mass lesions, which was approximately 84.8% to 90.2%. However, the sensitivity seemed lower than that of mass lesions, which was approximately 85.5% to 88.8%.^[10–12]^ Similar results were found when compared with BI-RADS. Sonoelastography had a higher pooled specificity (0.90 vs 0.68) but a lower sensitivity (0.69 vs 0.91) than BI-RADS, although there were no differences in AUC (0.8685 vs 0.9327) between them. One reason for the lower sensitivity was probably due to the high percentage of in situ lesions in the non-mass lesions. For example, there were 195 lesions of DCIS from the 491 malignant lesions in the 13 studies that provided detailed pathological results, occupying 39.71%. It was indicated that DCIS showed a significantly lower stiffness value than invasive ductal cancer in the literature.^[7]^ Another reason for the lower sensitivity may be the size. The mean diameters were no more than 30 mm in most of the studies. A smaller lesion size was associated with FN shear wave elastography results for malignant non-mass lesions in the literature.^[20]^ Interestingly, the size of the lesions included in Wang ZL et al’s study was the largest among these studies (mean diameter, 41.4 mm), but the sensitivity of sonoelastography in this study was only 0.58, significantly lower than the pooled sensitivity.^[21]^ This may be related to differences in histopathology and may indicate that sonoelastography is particularly suitable for breast non-mass lesions of a certain size range, rather than larger lesions being more easily diagnosed by this technique. The relationship between lesion size and sonoelastography performance still needs further research.

In recent years, more and more researchers have noticed the diagnostic value of elastography for breast non-mass lesions. Guo W et al established a predictive model for identifying the properties of breast non-mass lesions using conventional US, strain elastography (SE), and contrast-enhanced ultrasound (CEUS). Its sensitivity, specificity, positive predictive value, negative predictive value, and accuracy were calculated to be 98.0%, 94.0%, 94.3%, 97.9%, and 96.0%, respectively.^[26]^ Li S-Y et al analyzed the features of breast non-mass lesions on B-mode US, CEUS, and shear-wave elastography (SWE) and discovered that the combination of conventional US and SWE had the highest AUC of 0.935.^[14]^ Wang F et al explored the combined application of SWE and a novel blood flow imaging technique called Angio PLUS microvascular US imaging for diagnosing breast non-mass lesions and found an accuracy of 78.3% to 88.3%.^[27]^ These studies indicated that sonoelastography played a good complementary role on conventional US.

A similar meta-analysis about sonoelastography for breast non-mass lesions was reported in 2021.^[31]^ In comparison to the 2021 meta-analysis, our study had the following differences: First, the literature included in our study was different. Two articles were excluded: one because the main text was written in Italian,^[32]^ and the other because 2 studies were conducted in the same department,^[19,29]^ which may have some duplicate data, and the article with fewer lesions was excluded. Meanwhile, 5 new publications were included, with the corresponding author of one article providing the requisite data by email,^[22]^ while the other four were new studies in recent years. Second, given the widespread application of BI-RADS in clinical practice, we explored the differences between BI-RADS and sonoelastography, and the findings revealed that the latter had significantly higher specificity, which can be helpful to reduce misdiagnosis. Third, in the 2021 meta-analysis, meta-regression and subgroup analyses suggested that measurement indexes (quantitative or qualitative), the country of origin (China or others), and the number of lesions were possible sources of heterogeneity.^[31]^ However, statistical analysis indicated no heterogeneity arising from the threshold effect in our study. The Cochran *Q* test and the *I*^2^ test of DOR revealed no other heterogeneities. To be prudent, meta-regression was also performed in the above indexes in our study, but there were still no heterogeneities revealed, which may be for the different included articles between us.

There are some limitations in our study. First, few studies were included (i.e., fourteen). Second, unpublished data failed to be acquired, and language limitations might have affected the reliability of the results. Third, we did not analyze some factors that may influence image quality and overall diagnostic accuracy, such as pathological changes (e.g., calcification), race, breast thickness, lesion depth, and readers. Fourth, the value of sonoelastography combined with BI-RADS should be evaluated. However, that was only performed in 4 articles which showed a sensitivity of 0.61 to 0.97 and a specificity of 0.55 to 0.97.^[6,13,18,21]^ Therefore, it was not performed due to the few studies. Finally, this study was not registered, and there may be a risk of human bias.

## 5. Conclusions

In conclusion, this meta-analysis shows that sonoelastography has a higher specificity and a lower sensitivity for differential diagnosis between malignant and benign breast non-mass lesions compared with BI-RADS, although there were no differences in AUC between them. Effective application of sonoelastography can prevent the misdiagnosis of patients with benign lesions and avoid unnecessary medical treatment. More efforts should be made to combine these 2 methods and other technologies to evaluate if better accuracy can be achieved in the future.

## Acknowledgments

The authors thank Hao Sun for her help with statistics.

## Author contributions

**Conceptualization:** Hong Li.

**Data curation:** Ya-Na Yu.

**Formal analysis:** Peng Cong.

**Investigation:** Hong Li, Yun-Fei Zhang.

**Methodology:** Hong Li.

**Software:** Peng Cong.

**Validation:** Yun-Fei Zhang.

**Writing - original draft:** Hong Li, Peng Cong, Ya-Na Yu.

**Writing - review & editing:** Yun-Fei Zhang.
